# The influence of different proportions of rumen degradable protein and non-fiber carbohydrate consisted in feed ration on *in vitro* rumen fermentation, digestibility, gas production kinetics and enteric methane emission

**DOI:** 10.5455/javar.2025.l941

**Published:** 2025-08-18

**Authors:** Ujang Hidayat Tanuwiria, Mardiati Zain, Jasmal Ahmari Syamsu, Yunilas Yunilas, Andi Mushawwir, Yulianri Rizki Yanza

**Affiliations:** 1 Department of Animal Nutrition and Feed Technology, Faculty of Animal Sciences, Universitas Padjadjaran, Sumedang, Indonesia; 2 Department of Animal Nutrition and Feed Technology, Faculty of Animal Science, Andalas University, Padang, Indonesia; 3 Faculty of Animal Sciences, Universitity of Hasanuddin, Makassar, Indonesia; 4 Faculty of Agriculture, Department of Animal Science, University of Sumatera Utara, Medan, Indonesia

**Keywords:** Rumen fermentation, non-fiber carbohydrates, rumen degradable protein, *in vitro*, methane, gas kinetics

## Abstract

**Objective:**

The present study aimed to determine the influence of different rumen degradable protein (RDP)/non-fibrous carbohydrate (NFC) proportions on ruminal fermentation characteristics, gas production kinetics, and microbial populations.

**Materials and Methods:**

An *in vitro* batch culture trial was conducted using different combinations of RDP/NFC proportions categorized into six dietary treatments (*n* = 5 per treatment, three replicative runs). Combinations of balanced RDP/NFC proportions were 60% RDP: 35% NFC (P1, 1:3.65), 60% RDP: 40% NFC (P2, 1:4.17), 65% RDP: 35% NFC (P3, 1:3.37), 65% RDP: 40% NFC (P4, 1:3.85), 55% RDP: 39% NFC (P5, 1:5.06), and 55% RDP: 41% NFC (P6, 1:5.32).

**Results:**

The present study observed that the combination of a high proportion of RDP and NFC influenced *in vitro* rumen fermentation, such as volatile fatty acid and NH₃ concentrations, and *in vitro* organic matter digestibility. However, a high RDP (65%) with a low NFC (35%) positively influenced total gas production, gas kinetics, enteric methane production, and microbial population in the rumen.

**Conclusion:**

In this study, we revealed that the ratios of RDP and NFC in animal feed have a considerable impact on rumen fermentation, microbial population, and digestibility.

## Introduction

Nutritional feeding strategies have evolved to enhance ruminant productivity and economic efficiency. This evolution has mainly addressed the dietary balance and efficiency of principal components in feed, such as carbohydrates and proteins [1]. Although carbohydrate and protein requirements for ruminants have already been standardized, studies emphasizing optimizing feed source utilization to improve ruminant performance and productivity have been extended [2]. Accordingly, the current feeding strategy is significantly concerned with formulating feed rations to optimize utilizable nutrients by considering the critical role of microbial involvement in the rumen in degrading feed compounds [3].

Ruminant protein utilization is categorized into two components: rumen degradable protein (RDP), which is synthesized rapidly in the rumen, and rumen undegradable protein (RUP), which escapes rumen metabolism and is absorbed in the intestine [4]. Unlike RDP, RUP components, such as amino acids and other peptides, can pass rumen metabolism [5]. Thanks to rumen microbes, degraded feed proteins like RDP have turned into non-protein nitrogen in the rumen, like ammonia (NH_3_), that is utilized as a source for microbial growth and energy [6]. The crucial role of RDP and ruminal NH₃ further supports microbial protein synthesis (MPS) as a source of absorbable protein for ruminants to be absorbed in the intestine [7].

Notably, appropriate RDP can improve the efficiency of N utilization (ENU) in the rumen and consequently enhance the metabolic functions of animal health and deposition of nutritional quality of ruminant products, such as meat and milk [7]. However, excessive RDP would lead to N-inefficiency due to the exceeding ruminal NH₃ concentration, which is further absorbed into the blood, accumulated in the liver as urea, and then excreted in the urine [4].

Recent studies have indicated that certain dietary balances can enhance MPS, reduce nitrogen release rates, and synchronize with energy supply for rumen microbial growth [8]. Although microbial protein production in the rumen may improve, most of the *N* released as urinary urea leads to less efficient *N* utilization in ruminant metabolism [9]. Hence, an appropriate level of RDP is needed to improve the ENU and meet the metabolizable protein (MP) requirements for ruminants. Moreover, enhanced microbial protein by high RDP uptake can indirectly lead to increased methane production (CH_4_), whereas high digestible substrate may also elevate methanogen activity, which is responsible for CH_4_ in the rumen [10].

Carbohydrates are another major nutrient that ruminant production commonly depends on for forages and non-fiber carbohydrates (NFCs), such as concentrates or grains. Unlike forages, NFC can rapidly degrade in the rumen and support rumen microbial growth, which plays a crucial role in degrading fiber and protein [11]. Increasing NFC uptake enhances volatile fatty acid (VFA) production in the rumen, specifically propionate concentration, and consequently enhances the available energy for ruminant metabolism and production and decreases CH_4_ [12]. However, disproportionate NFC uptake may lower ruminal pH and lead to metabolic disorders, such as sub-acute ruminal acidosis, in the long term [2,13].

A feeding strategy is necessary to improve nutrient utilization by considering the protein and energy balance, such as the RDP and NFC proportion in the feed ration. Although both high RDP and NFC uptakes provide beneficial effects on rumen fermentation, the nutrient group mechanisms in the rumen are evidently contradictory to each other in ruminants. Hence, the critical aspect in improving ruminant production is maintaining a healthy ruminant while efficiently reducing the environmental impact, where most rumen microbial species and genera depend on these two sources [11]. Evidence is needed to corroborate the appropriate proportion of a combination of RDP and NFC in feed ration and their effects on the rumen fermentation profile, methane emission, and nutrient digestibility rates. Therefore, this study aimed to determine the optimal proportion of various combinations of RDP and NFC in a feed ration and their influence on rumen fermentation, feed digestibility, gas production, and kinetics, and enteric CH_4_ through an *in vitro* study.

## Materials and Methods

### Ethical approval

The methodology for the present *in vitro* experiment was approved by the Padjadjaran University Research Committee. There is no need for any ethical approval, as no living animals were harmed or used in these *in vitro* trials.

### Experimental design, substrate, and treatments

Preparation of the *in vitro* study was obtained in the Laboratory of Ruminant Nutrition and Feed Chemistry, Department of Animal Nutrition and Feed Technology, Faculty of Animal Science, Universitas Padjadjaran. The present study used a combined diet composed of forage bases such as elephant grass and Indigofera and several agricultural by-products such as corn stalks, rice straw, corn husks, ground corn, cassava, soybean meal, coconut meal, palm meal, and tofu dregs with varying chemical compositions. Feed sources were dried at 60°C for 24 h before being milled into 1 mm particle size, then their chemical composition following AOAC [14] analysis protocol, such as dried matter (DM; no. 934.01), ash (no. 942.05), crude fiber (CF; no. 978.10), crude protein (CP; 954.01), and ether extract (EE; no. 973.18), while RDP and NFC of each feed source were determined through the Tilley et al. [15] method. The percentage protein loss of the incubated substrate was measured as protein degraded in the rumen (RDP), while NFC is calculated using the following formula: NFC = 100 - neutral detergent fiber - CP - EE - ash. Information concerning the chemical composition of feed materials used in the current experiment is listed in Table 1.

Treatments used in the present study were grouped following the proportion of RDP and NFC, which consisted of dietary ratio (Table 2). Each ration treatment was mixed and formulated from the listed sources. All treatments were balanced to the 65% total digestible nutrient (TDN) value, with different RDP and NFC proportion combinations in each treatment. The combination of balanced RDP to NFC proportions ratio was 60% RDP: 35% NFC (P1, 1:3.65), 60% RDP: 40% NFC (P2, 1:4.17), 65% RDP: 35% NFC (P3, 1:3.37), 65% RDP: 40% NFC (P4, 1:3.85), 55% RDP: 39% NFC (P5, 1:5.06), and 55% RDP: 41% NFC (P6, 1:5.32). The diet balanced with 55%, 60%, and 65% of RDP comprised 77, 96, and 104 gm/kg DM in the diet, respectively. Meanwhile, 35%, 40%, and 41% of NFC consisted of about 350, 400, and 410 gm/kg in the diet.

**Table 1.** Nutrient composition of diet sources used in the *in vitro* experiment.

Feed source	Nutrient composition (gm/kg DM)							
	DM	Ash	CF	CP	EE	TDN^*^	RDP^**^	NFC^***^
Elephant grass	929.5	130	308.6	130	26.1	571.7	71.3	42.2
Corn stalks	939.8	80.1	16.5	112.3	16.5	545.3	52.9	78.4
Rice straw	941.2	196.9	13.6	38	13.6	310.7	18.3	1.3
Corn husks	869.4	37.1	284.9	70.8	17.6	534.9	31.4	167.2
Indigofera	896.3	85.9	174.3	309.2	23.9	675.6	225.9	348.7
Grounded corn	884.7	113.1	16.3	152.4	29.8	810.9	115.8	704.7
Cassava	892.8	35.3	30.9	32.3	19.5	676.9	15.9	912.9
Soybean meal	890	69.9	27.7	492.1	92.1	902.1	147.1	345.9
Coconut meal	935.5	78.9	131	78.5	145.9	793.2	54.5	696.7
Palm meal	962.8	36.7	313.2	193.4	90.8	616.1	116.1	679.1
Tofu dregs	936	25.7	214.3	203.8	21.4	694.9	151.0	758.4

CF = crude fiber, CP = crude protein, DM = dried matter, EE = ether extract, NFC = non-fiber carbohydrate, RDP = rumen degradable protein, TDN = total digestible nutrient.

**Table 2.** Feed ingredients and chemical composition of dietary treatments used in the *in vitro* experiment.

Feed source	Feed formulation among treatments (gm/kg DM)					
	P1	P2	P3	P4	P5	P6
Feed ingredients						
Elephant grass	50	-	330	298.9	135.4	120
Corn stalks	243.4	207.5	-	-	132.2	124.4
Rice straw	86.9	141.9	86.2	81.8	63.6	66.8
Corn husks	50	-	-	-	132.2	124.4
Indigofera	150	140	185.3	130	26	23.7
Grounded corn	236.4	169	350	285.1	118.6	113
Cassava	-	17.7	-	-	103.6	120
Soybean meal	43.7	67.5	-	-	82.6	82.9
Coconut meal	121.3	173.6	35.3	37.3	156.3	149.6
Palm meal	-	-	-	50	15	29.9
Tofu dregs	8.3	72.9	-	106.9	24.4	33.6
Mixed mineral	10	10	10	10	10	10
Chemicals composition (gm/kg DM)						
DM	914.2	920.1	909.7	916.7	912.7	913.4
Ash	97.4	96.6	118.6	105.9	84.2	82.3
CP	160	160	160	160	140	140
CF	167.2	164	171.5	187.7	174.9	173.5
EE	39.9	47.2	29.9	32.8	47.4	47.5
OM	533.8	522.8	514.3	508.1	554.7	557.4
Potential degrading nutrient (%)						
TDN	65	65	65	65	65	65
RDP	60	60	65	65	55	55
NFC	35	40	35	40	39	41

CF = crude fiber, CP = crude protein, DM = dried matter, EE = ether extract, NFC = non-fiber carbohydrate, RDP = rumen degradable protein, TDN = Total digestible nutrient.

The *in vitro* experiment was conducted through batch culture incubation following the Theodorou et al. [16] technique following the modified protocol prepared by Yanza et al. [17] with some development. In brief, about 500 mg of dietary ration and 50 ml of mixed buffered rumen fluid were used in each bottle and fermented in 100 ml bottles for 24 h. The study was performed in a 6 × 5 (treatment × bottle) experimental design, trialed in triplicates on three consecutive days (one replicate was done for one day), followed by two bottles consisting of buffered rumen fluids with no dietary treatments as blank samples.

### Preparation and rumen in vitro batch culture incubation

Fresh rumen fluid was collected from a slaughterhouse of six commercial Brahman Angus bulls (two different bulls were slaughtered for each incubation), whereas cattle had been previously fed with commercial total mixed ratio (TMR) diets. Rumen fluid from each cattle was taken from the upper, middle, and lower parts of the rumen and filtered through four layers of cheesecloth into a 1.5 l vacuumed flask maintained at 39°C. Those vacuumed flasks filled with rumen fluid were then transferred to the laboratory, towed at a 39°C water bath, and mixed with the previously prepared McDougall buffer. Approximately 400 ml of rumen fluids from both vacuum flasks were mixed in a 2 l glass beaker and diluted with 1,600 ml of McDougall buffer (9.8 gm NaHCO₃, 4.65 gm Na_2_HPO_4_·2H_2_O, 0.57 gm KCl, 0.47 gm NaCl, 0.12 gm MgSO_4_·7H_2_O, and 0.04 gm CaCl_2_ per liter of buffer). The mixed buffered rumen fluid flowed with CO_2_ gas before each 50 ml of buffered rumen fluid was transferred into the 100-ml fermenter bottle filled with a 500 mg dietary experimental ration. Moreover, the bottle was sealed with rubber and brass sealer and put into a batch incubator set to a 39°C temperature. After 24 h of incubation, the fermented bottles were then opened, and buffered rumen fluid from each bottle was prepared for analysis, such as rumen fermentation profile, microbial population, and digestibility after 24 h of incubation.

### Analysis of microbial population, rumen fermentation profile, and digestibility

Rumen pH was measured directly after the opened fermenter bottle using a pH meter (Hanna Instruments, HI98191, Romania). The supernatant in each fermenter bottle was poured into a 50 ml Falcon flask that was arranged for collecting samples before analysis. Approximately 1 ml of buffered rumen fluid supernatant was collected for counting the protozoa under a light microscope (Zeiss, type Primo Star no. 5, Jena, Germany). Protozoa were counted using a drop of fermented ruminal fluid (1 ml) and 3.7 % formalin (6 ml) using a similar microscope.

The protozoa were counted according to the protocol described by Yanza et al. [17] with modified and defined measurements, 10 µl for *Entodiniiae* and 100 µl for *Isotricha* and *Duplodiniiae*. Meanwhile, the total bacteria population was obtained using Thoma chambers (0.02 mm depth, BlauBrand, Wertheim, Germany) using a 20 µl drop of fermented ruminal fluid and 6,980 µl of Hayem solution. Approximately 10 µl of mixed buffered rumen fluid-Hayem solution was put in the arranged Thoma chambers covered with glass and microscopically counted as Cieslak et al. [18] showed. Some amounts of the supernatant were collected for NH₃ concentration analysis through Conway et al. [19] and for the VFA analysis gas chromatography (GC-14A, Shimadzu Corporation, Japan, Tokyo) fitted with a flame ionization detector (FID). The remaining supernatant was added with three drops of HgCl_2_ and then incubated for 24 h (39°C) in a water bath and filtered through filter paper to determine the digestibility rate from the fermentation residue [17]. The percentage of weight loss of the incubated substrate after correction of residual weight and blank was measured as digestibility.

### Measurement of total gas production (TGP), enteric methane, and gas kinetics

The gas production was measured every 2 h using a 10 ml syringe with a 0.1 mm needle injected into the rubber part of the sealed bottle during 24 h *in vitro* incubation and collected into the vacuumed and sealed 100 ml bottle. The 24-h recording of each bottle of gas production was summarized for the TGP, while the gas kinetics data were analyzed following an exponential formula [20]. Moreover, about 5 ml of the total gas was taken and collected in a 5 ml Vacutainer for methane concentration measurement. The methane analysis used the Shimadzu 8A GC with a FID following the Haryati et al. [21] procedure.

### Statistical analysis

The gas kinetics data from each sample was analyzed statistically through the mathematical model described by Ørskov et al. [20] as follows:

*p* = *a* + *b*(1 − *e*−*ct*)

where *p* is gas accumulation at the t-period; a is gas production of the rapidly degraded fraction; b is gas production of the slowly degraded fraction; e is the exponential factor; c is the coefficient value; and t is the period of fermentation at time (h).  All data were analyzed using SPSS (v. 29).

All data were then statistically analyzed through one-way ANOVA using PROC MIXED procedures of SAS software (SAS on demand for academics, online version), in which dietary groups were considered a fixed factor and days of incubation were considered a random factor. Moreover, means between groups were calculated through the LSMEANS protocol, and the SEM value was also shown in each analyzed parameter. Once dietary groups in an observed parameter were obtained (*p* < 0.05 or 0.05 < *p* < 0.10), between-group differences were declared as significant or tending to be differences, respectively, followed by the Tukey post-hoc test to determine the range of differences between experimental groups.

## Results

In the *in vitro* study, we investigated the effect of different dietary groups on organic digestibility rate, microbial populations, and gas production during a 24-h incubation. The results showed that the P3 and P4 dietary groups had significantly higher organic digestibility rates (IVOMD) than the P2 and P5 groups (*p* = 0.01; Table 3). However, all dietary groups had similar *in vitro* dry matter digestibility rates and pH values. Although it showed similar values to P3 and P4, the P1 group also showed a higher IVOMD rate than the P5 group. Moreover, the P1, P2, P3, and P4 groups obtained a higher ruminal fluid NH₃ concentration than the P5 group (*p* < 0.01). A lower NH3 concentration in ruminal fluid was also shown in the P6 group compared to the P1, P2, and P3 groups but had no significant difference with the P4 dietary group. There is no significant difference between experimental groups concerning VFA proportions such as acetate, propionate, iso-butyrate, butyrate, iso-valerate, and valerate. The acetate:propionate ratio also revealed no significant differences. However, the P1 and P4 groups produced the highest total VFA concentration in the fermented ruminal fluid compared to the P2 and P5 dietary groups but similar to the P1 group (*p* < 0.01). Moreover, P4 had a higher VFA concentration than the P3 and P6 dietary groups.

Concerning the microbial populations (Table 4), the P1 dietary group obtained the highest population, and the P5 dietary group received the lowest (*p* < 0.01; Table 4). However, the P3 group was higher than P5 and P6, but the P2 and P4 groups were only more elevated than the P5 groups. Meanwhile, no significant difference was obtained between dietary groups concerning total protozoa and the Entodiniiae population. However, the Isotricha and Duplodiniiae populations were higher in the P3 group than in all other dietary groups (*p* < 0.01). Moreover, the P1 and P4 groups had higher Holotricha populations than the P2 and P6 groups, and the Duplodinium population of the P1 and P4 groups was higher than that of the P2 group.

The gas production of each dietary group was recorded every two hours during the 24-h incubation period. The results (Table 5) showed that the TGP significantly varied among dietary groups, with the P6 group showing the highest TGP, while the P1 and P2 groups had the lowest (*p* < 0.01). Similarly, when expressed as TGP/*in vitro* DM digestibility (IVDMD) and TGP/IVOMD, the P6 group had the highest gas production, while the P1 and P2 groups had the lowest. However, the P3 and P4 groups showed varied results (*p* < 0.01). The P3 and P6 groups were higher than the P6 group when expressed as TGP/DM substrate (DMs), while the P1 and P2 groups had the lowest (*p* < 0.01). There were no significant differences concerning the CH_4_ concentration expressed in mM. However, the P3 and P4 groups were significantly lower in methane expressed in CH_4_/TGP (*p* = 0.02) and tended to be lower in CH_4_/IVDMD (*p* = 0.06) and CH_4_/IVOMD (*p* = 0.07) than the P1 and P2 groups.

**Table 3.** Rumen fermentation profile after 24h fermentation *in vitro*.

Observed parameters	P1	P2	P3	P4	P5	P6	SEM	*p* value
*In vitro* digestibility								
IVDMD (%)	66.64	64.76	69.65	68.77	65.07	66.92	0.99	0.16
IVOMD (%)	72.21^ab^	68.90^bc^	73.22^a^	72.84^a^	67.26^c^	70.40^abc^	0.58	0.01
Fermentation profile								
pH	6.98	6.98	6.97	6.95	6.98	6.97	0.01	0.14
NH_3_ (mM)	6.20^a^	6.00^a^	6.31^a^	5.74^ab^	4.47^c^	4.78^bc^	0.22	<0.01
Total VFA (mM)	163.0^ab^	127.0^c^	158.2^b^	184.0^a^	134.1^c^	160.2^b^	4.05	<0.01
VFA proportion (%)								
Acetate	55.82	64.49	64.65	65.36	63.60	61.16	1.92	0.72
Propionate	23.38	19.03	20.04	20.35	18.80	19.50	0.84	0.68
Iso-butyrate	2.22	1.76	2.07	1.537	1.902	1.86	0.17	0.78
Butyrate	13.3	11.1	9.77	9.81	12.12	13.5	0.75	0.55
Iso-valerate	3.28	2.41	2.32	1.939	2.441	2.78	0.24	0.53
Valerate	2.03	1.31	1.22	1.069	1.201	1.29	0.14	0.35
Acetate: Propionate	2.61	3.8	3.41	3.425	3.783	3.32	0.24	0.77

IVDMD: *in vitro* dried matter digestibility; IVOMD = *in vitro* organic matter digestibility; NH_3_: ammonia production; VFA: volatile fatty acids. ^abc^ Means with different superscript letter in the same column showed statistically different at *p* < 0.05.

**Table 4.** Rumen microbial population after 24 h fermentation *in vitro*.

Observed parameters	P1	P2	P3	P4	P5	P6	SEM	*p* value
Microbial population								
Bacteria population(10^8^/ml)	26.38^a^	17.23^bc^	18.5^b^	16.14^bc^	12.51^d^	13.81^cd^	1.39	<0.01
Protozoa population (10^3^/ml)	89.95	98.37	99.01	100.10	103.20	93.45	5.61	0.17
*Isotricha* (10^3^/ml)	0.09^ab^	0.03^c^	0.12^a^	0.07^b^	0.04^bc^	0.02^c^	0.01	<0.01
*Duplodiniiae *(10^3^/ml)	1.22^b^	0.96^c^	1.63^a^	1.39^b^	1.09^bc^	1.15^bc^	0.04	<0.01
*Entodiniiae *(10^3^/ml)	88.6	97.4	97.3	98.63	102.1	92.3	5.60	0.16

^abc^Means with different superscript letter in the same column showed statistically different at *p* < 0.05.

**Table 5.** Gas production and kinetics after 24h rumen fermentation *in vitro*.

Observed parameters	P1	P2	P3	P4	P5	P6	SEM	*p* value
Gas production								
TGP (ml)	67.26^c^	69.87^c^	82.69^ab^	82.01^ab^	77.15^b^	84.56^a^	1.16	<0.01
TGP/ DMs (ml/gm)	148.9^c^	146.5^c^	177.1^a^	172.9^ab^	164.8^b^	180.9^a^	2.37	<0.01
TGP/IVDMD (ml/gm)	223.9^c^	228.2^c^	248.4^bc^	252.4^abc^	277.8^ab^	282.2^a^	5.31	<0.01
TGP/IVOMD (ml/gm)	214.6^bc^	206.6^c^	238.7^b^	239.5^b^	241.0^b^	270.4^a^	5.56	<0.01
CH_4_ (mM)	7.38	7.64	7.60	7.44	7.74	8.40	0.13	0.14
CH_4_/TGP (mM/ml)	0.051^a^	0.055^a^	0.044^b^	0.043^b^	0.047^ab^	0.047^ab^	0.001	0.02
CH_4_/DMs (mM/gm)	15.86	15.96	16.34	15.68	16.48	17.97	0.29	0.12
CH_4_/IVDMD (mM/gm)	24.57^AB^	26.28^AB^	23.90^B^	23.33^B^	27.52^A^	27.76^A^	0.76	0.06
CH_4_/IVOMD (mM/gm)	22.49^B^	23.93^AB^	22.61^B^	21.62^B^	23.72^AB^	26.08^A^	0.51	0.07
								
Gas kinetics								
*a *	−0.58	−2.20	−1.94	−0.96	−1.25	−1.30	5.61	0.25
*b *	177.0^ab^	117.8^c^	212.0^a^	164.8^abc^	129.1^bc^	152.7^bc^	0.26	0.01
*a + b*	176.4^ab^	115.8^c^	210.0^a^	163.4^abc^	127.8^bc^	143.7^bc^	10.2	0.01
*c *	0.11	0.06	0.03	0.04	0.05	0.05	9.74	0.29

*a = *gas produced immediately by soluble fraction*, a+b = *potential extent of gas production*, b = *gas produced by insoluble fraction*, c = *constant rate of gas production of insoluble fraction, DMs = dried matter substrate, CH_4_ = methane production, IVDMD = *in vitro* dried matter digestibility, IVOMD = *in vitro* organic matter digestibility, TGP = total gas production.^abc^Means with different superscript letter in the same column showed statistically different at *p *< 0.05.

Additionally, the produced gas of soluble fraction (a) and constant rate of insoluble fraction (c) of gas kinetics parameters of incubated ruminal fluid among dietary groups were not significantly different. However, the produced gas of insoluble fraction (b) and the potential extent of gas production (a+b) showed the highest results in the P3 group compared to the P2, P5, and P6 groups, where the P1 group was also higher than the P2 group (*p *= 0.01).

## Discussion

### Digestibility, rumen fermentation profile, and microbial population

A feeding strategy that balances energy and protein utilization is critical to improving feed utilization efficiency while enhancing ruminant productivity. The primary digestive process in ruminants involves microbes in the rumen, a chamber where fermentation is initiated. Ingested feed nutrients in the rumen are broken down from large particles into smaller particles; hence, available nutrients may rapidly integrate into further metabolic processes [22]. NFC and RDP are known for their readily and rapidly degraded nutrients in the rumen and discharged readily available nutrients; either can be utilized to support ruminal microbes‘ perseverance during the fermentation process or provide certain fermentation products such as NH₃ from protein and VFA, mostly from carbohydrates [11].

Although the TDN of each trialed dietary group was balanced at 65%, the RDP and NFC proportions among dietary groups were varied and showed a different efficiency in degrading organic compounds during fermentation. In the present study, the organic particles of the P1, P3, and P4 dietary groups were efficiently digested. The increased organic digestibility to those dietary groups also aligned with the enhanced NH_3_ and VFA concentration. However, ruminal pH values among dietary groups were similar. It indicates that the various combinations of RDP and NFC proportion were maintained in the ruminal environment during fermentation, which is essential for microbial growth activity [23].

The NH₃ concentration ranged from approximately 4.47–6.20 mM. The present NH_3_ results were lower than the Rosmalia et al. [11] findings, who had also studied the balance proportion of RDP (50%–60%) and NFC (30%–40%) and obtained approximately 7.59–8.09 mM NH_3_ concentration. The present NH_3_ results for P1, P2, P3, and P4 dietary groups were still in the normal ranges, although P5 and P6 groups produced lower than the recommended value (<5 mM). The high RDP proportion increases nitrogen availability in the rumen, which is strongly associated with increased MPS. Unfortunately, MPS was not observed in the present study. Notwithstanding the evidence, the enhanced NH₃ concentration in the present research emphasizes the value of nitrogen availability in microbial growth and fermentation processes [24].

The highest VFA production in the P4 group was noticed as the combination of high RDP and NFC diets (65% and 40%, respectively). Moreover, although lower than the P4, the P1, P3, and P6 dietary groups also had higher VFA concentrations than others (P2 and P5 groups). This evidence implies that a higher RDP and NFC combination modulated ruminal microbial activity more efficiently and enhanced VFA concentration [25]. This finding was aligned with Putri et al. [8], who suggested that a proper RDP and NFC balance may optimize nutrient utilization by ruminal microbes during fermentation. However, there is a similarity in partial VFA proportion and A:P ratio among dietary groups. Various dietary compositions by different feed sources in the TMR also need to be considered because of different modes of action in stimulating different microbial genera.

On the contrary, the highest total bacteria population was shown by the P1 dietary group (60% and 35% of RDP and NFC, respectively), regardless of the similar P2, P3, and P4 total bacteria populations that were still higher than the P5 and P6 dietary groups. This result may indicate that various RDP and NFC proportions lead to changes in the total VFA production without necessarily altering the relative proportions of individual VFAs (acetate, propionate, butyrate) or the A:P ratio. However, no MPS was observed in the present study. Nevertheless, a similar pH value indicates a stable rumen environment among treatments, supporting the ruminal microbes to optimize the fermentation efficiency in producing individual VFAs [26].

The optimum microbial activity was shown by a combination of high RDP and NFC -proportioned dietary groups regarding their IVOMD fermentation rate, NH₃, and VFA production. It can be suspected that high NFC and RDP uptake can stimulate rumen microbes to enhance fibrous and protein digestion in the rumen as well as provide the available N for rumen microbial growth; thus, rumen microbes such as protozoa and bacteria populations in the present study were increased [27].

An appropriate combination of high RDP and NFC may improve the ENU by using dietary carbohydrates as a fermentable energy source for diverse ruminal microbes. Thus, the ruminal fermentation of carbohydrates is adapted to ruminal protein degradation [4]. Our findings may relate to Putri et al‘s [28] results, which reported an enhancement in VFA concentration (62%), NH_3_ concentration (161%), and rumen digestibility (28%) after 24 h *in vitro* batch culture fermentation by the increased proportion of NFC (65%–70%) and RDP (55%–65%). Hence, non-structural carbohydrates and available nitrogen were modified during fermentation in the rumen and enhanced VFA production, such as acetate and propionate production, through the degradation of carbohydrate structures. However, no specific bacteria were observed in the present study.

Even though it is worth noticing that diverse microbial species, including cellulolytic and proteolytic bacteria, grow in the rumen, contributing to energy and protein degradation. For instance, *Butyrivibrio fibrisolvens*, *S. bovis*, and other cellulolytic bacteria, as well as the protozoa, deteriorate plant polysaccharides and proteins to produce VFA, peptides, and NH₃ [29]. Furthermore, produced NH₃ and peptides provide essential nutrients for other rumen bacteria genera for biosynthesis and growth by assimilating available nitrogen through glutamate and glutamine pathways, synthesizing them, and determinately increasing the microbial biomass [30]. These metabolic pathways provide synergistic action between microbial species and genera that may improve nutrient utilization in the rumen.

In the present study, various combinations of NFC and RDP proportion had a lesser impact on total protozoa populations, neither in Entodiniiae protozoa. However, the highest activity was determined in specific protozoa, i.e., Isotricha and Duplodiniiae, emphasized by the selective influence of P3 dietary groups with a combination of high NFC (65%) and lower RDP (35%). Such evidence may indicate that the high NFC proportion influenced the increased Isotricha and Duplodiniiae populations in the ration, in which those microbes constrained the degradation activity of feed components consisting of highly degradable carbohydrates [31].

### Gas production, gas kinetics, and enteric CH4

**Figure 1.**
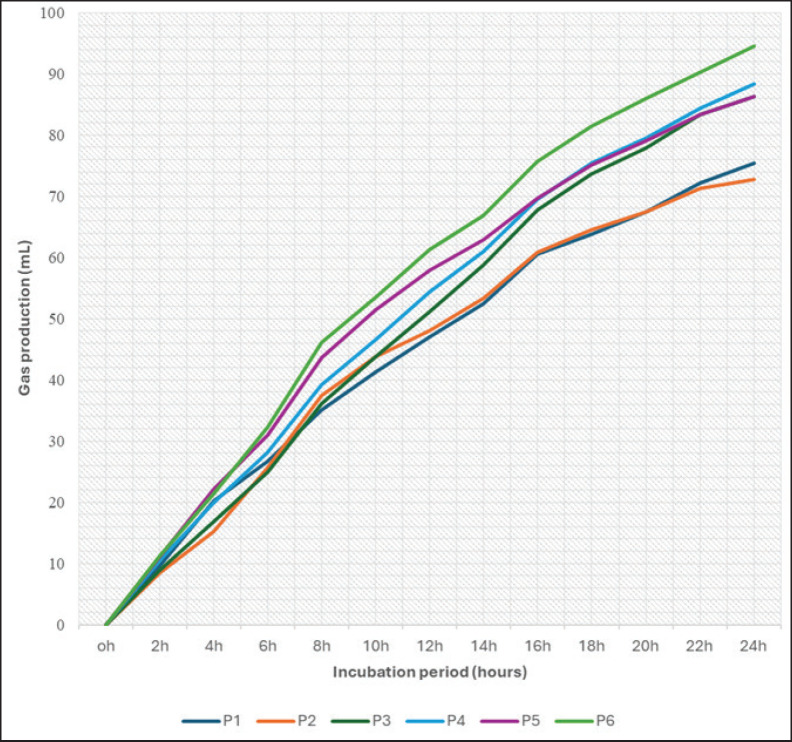
Gas fermentation flows from different RDP to NFC ratio of 24 h *in vitro* incubation.

The gas production (Table 5) was also depicted with gas production dynamics in Figure 1 that may reflect the influence of the dietary combination of RDP and NFC proportion on rumen fermentation kinetics and gas output. Combinations of RDP and NFC proportion from the P6 dietary group exhibited the highest TGP (TGP/DMs) compared to others, notified as the lowest RDP and the highest NFC (55% and 41%, respectively). Meanwhile, the lowest gas production was found in the P1 dietary group, with higher RDP than P6 (60% and 35%) and P2 (60%:40%). The present results indicated that the different NFC proportions in the ration could affect the various gas productions. As the gas production indicates the pattern of feed fermentation by rumen microbes, enhanced readily degradable carbohydrates also align with the increased gas production in the rumen [32]. Additionally, the balance between the RDP and NFC content could increase protein degradation by microbes, thereby promoting microbial growth [11,25]. Such evidence was also alienated from the present gas kinetics (a+b) results on similar dietary groups but did not significantly affect the gas production rate (c). Differences in gas kinetics could also be indicated by the modulation of rumen microbial activity, leading to changes in the digestibility value of various easily soluble fractions in the feed [33]. However, the existence of easily soluble carbohydrates that would be rapidly converted into gas, increasing TGP, might also represent the increased methanogenesis process by rumen microbes in yielding CH_4_ during fermentation [18]. Enteric CH_4_ may rise due to the increased NDF and hemicellulose content as it alters the proportion of acetic acid, which produces hydrogen (H_2_) as a substrate in the methanogenesis reaction [34].

In the present study, dietary groups with high RDP (65%), such as the P3 and P4 groups, showed an inhibition of CH_4_ when expressed in CH_4_/TGP, CH_4_/IVDMD, and CH_4_/IVOMD. However, the P1 dietary group also showed a lower enteric CH_4_ when expressed as CH_4_/IVOMD. This evidence suggests that the fibrous and carbohydrate components rapidly degrade in the rumen but do not directly go through the methanogenesis pathways. Thus, enteric CH_4_ was inhibited.

Degraded fibrous and carbohydrate compounds mostly involve fibrinolytic bacteria, protozoa, and methanogenic archaea, in which converted feed cell wall polysaccharides into VFAs are also followed by the production of CO_2_ and free H_2_ in the rumen. Further, the free H_2_ is primarily utilized by methanogenic bacteria to produce CH_4_ [35]. In the present study, the presence of high RDP inferred methanogenesis through rumen fermentation and microbial interactions. Accordingly, *in vitro*, CH_4_ was reduced by approximately 23% when expressed as CH_4_/TGP and reduced by approximately 15% and 17% when expressed as CH_4_/IVDMD and CH_4_/IVOMD, respectively. Previous studies confirmed that a high RDP proportion in feed rations is committed to enhancing the growth and activity of certain rumen microbes that utilize NH₃ more efficiently for protein synthesis [28]. This process can lead to a shift in fermentation end products, where free H_2_ is shifted into the propionic production pathway, potentially favoring production over acetate [36]. Hence, the limited availability of free H_2_ consequently diminished the activity of methanogens in producing CH_4_ [37].

However, no methanogens were observed in the present study. Nevertheless, the increase in rumen-degradable protein can stimulate the growth of specific microbial populations that are less methane-producing or even methane-consuming. Sahroni et al. [38] found that rations with a higher RDP ratio increased the population of bacteria and protozoa while providing a higher supply of VFAs, demonstrating how RDP influences fermentation pathways and end-product formation. Thus, alterations of microbial activity in the rumen ecosystem, driven by the availability of different nitrogen sources, could decrease methane emissions.

Nevertheless, although high RDP (65%) alters ruminal NH₃ production, the variability in dietary composition may have influenced the dynamics of rumen microbes efficiently utilizing nitrogen [39]. The absence of results such as MPS and methanogen populations in the present study may limit the current understanding of specific microbial interactions regarding the influence of RDP and NFC on the dynamics of rumen microbial activity and methanogenesis processes. Therefore, it is recommended that future studies assess the dynamic rumen environment by incorporating an *in vivo* approach to validate the interaction between the balance of RDP and NFC with rumen microbial dynamics, potentially leading to more effective strategies for mitigating methane emissions while improving ruminant production.

## Conclusion

It can be concluded that a combination of high RDP (65%) and NFC (40%) proportions influences *in vitro* rumen fermentation parameters such as total VFA and NH_3_ concentration. The dietary combination of high RDP and NFC proportion also positively affected total bacteria and protozoa activity, particularly *Isotrichae* and *Duplodiniiae*, consequently increasing the organic digestibility. Although gas production and various results of gas kinetics were found in the present *in vitro* study, ruminal enteric CH_4_ was reduced when dietary feed consisted of a combination of high RDP with low NFC proportion (65% of RDP and 35% and 40% of NFC, respectively).

## List of abbreviations

ANOVA, Analysis of Variance; AOAC, Association of Official Analytical Collaboration; CF, crude fiber; CP, crude protein; DM, dried matter; DMs, dried matter substrate; EE, ether extract; ENU, efficiency of n utilization; FID, flame ionization detector; CH_4_, methane production; IVDMD, *in vitro* dried matter digestibility; IVOMD, *in vitro* organic matter digestibility; LSMEANS, Least Squares Means MP, Metabolizable Protein; MPS, microbial protein synthesis; NFC, non-fiber carbohydrates; NH_3_, ammonia; PROC MIXED, Procedure MIXED; RDP, rumen degradable protein; RUP, rumen undegradable protein; TDN, total digestible nutrient; TGP, total gas production; TMR, total mixed ratio; VFA, volatile fatty acids.

## References

[1] Yousefinejad S, Fattahnia F, Kazemi-Bonchenari M, Nobari B, Ghaffari MH (2021). Effects of protein content and rumen-undegradable to rumen-degradable protein ratio in finely ground calf starters on growth performance, ruminal and blood parameters, and urinary purine derivatives. J Dairy Sci.

[2] Erickson PS, Kalscheur KF (2020). Nutrition and feeding of dairy cattle. Animal agriculture: sustainability, challenges and innovations.

[3] Su M, Chen D, Zhou J, Shen Q (2022). Effects of different dietary carbohydrate sources on the meat quality and flavor substances of xiangxi yellow cattle. Animals.

[4] Savari M, Khorvash M, Amanlou H, Ghorbani GR, Ghasemi E, Mirzaei M (2018). Effects of rumen-degradable protein: rumen-undegradable protein ratio and corn processing on production performance, nitrogen efficiency, and feeding behavior of Holstein dairy cows. J Dairy Sci.

[5] Stojanovic B, Simic A, Grubic G, Djordjevic N, Bozickovic A, Davidovic V (2019). Protein degradability of grassland forage under simulated rotational spring grazing. J Agric Sci.

[6] Ma SW, Faciola AP (2024). Impacts of slow-release urea in ruminant diets: a review. Fermentation.

[7] Valizadeh A, Kazemi-Bonchenari M, Khodaei-Motlagh M, Moradi MH, Salem AZM (2021). Effects of different rumen undegradable to rumen degradable protein ratios on performance, ruminal fermentation, urinary purine derivatives, and carcass characteristics of growing lambs fed a high wheat straw-based diet. Small Rum Res.

[8] Putri EM, Zain M, Warly L, Hermon H (2019). *In vitro* evaluation of ruminant feed from West Sumatera based on chemical composition and content of rumen degradable and rumen undegradable proteins. Vet World.

[9] Clemmons BA, Voy BH, Myer PR (2019). Altering the gut microbiome of cattle: considerations of host-microbiome interactions for persistent microbiome manipulation. Microb Ecol.

[10] MikoŁAjczyk K, Pecka-KieŁ B E, Zachwieja A (2020). Methanogenesis and synthesis of volatile fatty acids in the rumen of cows and their changeability under the influence of ensiled plant additives. Folia Pomer Univ Technol Stetin Agric Aliment Pisc Zootech.

[11] Rosmalia A, Permana IG, Despal D, Toharmat T, Pambudi FR, Arif SIZ (2023). Effect of dietary non-fiber carbohydrate sources and sulfur supplementation on *In vitro* ruminal fermentation and digestibility of the dairy ration. Iran J Appl Anim Sci.

[12] Oliveira JPP, Bicalho AF, Malacco VMR, Lage CFA, Saturnino HM, Coelho SG (2020). Supplementation with different non-fiber carbohydrate sources in dairy cow diets with high or low rumen-undegradable protein content. Arq Bras Med Vet Zootec.

[13] Chen Q, Cui YF, Zhang ZX, Jiang FC, Meng XY, Li JJ (2024). Effect of alfalfa supplementary change dietary non-fibrous carbohydrate (NFC) to neutral detergent fiber (NDF) ratio on rumen fermentation and microbial function in Gansu alpine fine wool sheep (*Ovis aries*. Anim Biotechnol.

[14] AOAC (2007). Official methods of analysis (18th ed.).

[15] Tilley JMA, Terry RA (1963). A two-stage technique for the *in vitro* digestion of forage crops. Grass Forage Sci.

[16] Theodorou MK, Williams BA, Dhanoa MS, McAllan AB, France J (1994). A simple gas production method using a pressure transducer to determine the fermentation kinetics of ruminant feeds. Anim Feed Sci Technol.

[17] Yanza YR, Szumacher-Strabel M, Bryszak M, Gao M, Kolodziejski P, Stochmal A (2018). *Coleus amboinicus* (Lour.) leaves as a modulator of ruminal methanogenesis and biohydrogenation *in vitro*. J Anim Sci.

[18] Cieslak A, Zmora P, Matkowski A, Nawrot-Hadzik I, Pers-Kamczyc E, El-Sherbiny M (2016). Tannins from sanguisorba officinalis affect *in vitro* rumen methane production and fermentation. J Anim Plant Sci.

[19] Conway EJ, O'Malley E (1942). Microdiffusion methods. Ammonia and urea using buffered absorbents (revised methods for ranges greater than 10 μg. N). Biochem J.

[20] O̊rskov ER, McDonald I (1979). The estimation of protein degradability in the rumen from incubation measurements weighted according to rate of passage. J Agric Sci.

[21] Haryati RP, Jayanegara A, Laconi EB, Ridla M, Suptijah P (2019). Evaluation of chitin and chitosan from insect as feed additives to mitigate ruminal methane emission. AIP Conf Proc.

[22] Nagaraja TG, Millen D, De Beni Arrigoni M, Lauritano Pacheco R (2016). Microbiology of the rumen. Rumenology.

[23] Hall MB (2017). Nitrogen source and concentration affect utilization of glucose by mixed ruminal microbes in vitro. J Dairy Sci.

[24] Lee C, Morris D L, Copelin J E, Hettick J M, Kwon I H (2019). Effects of lysophospholipids on short-term production, nitrogen utilization, and rumen fermentation and bacterial population in lactating dairy cows. J Dairy Sci.

[25] Rosmalia A, Permana IG, Despal D (2022). Synchronization of rumen degradable protein with non-fiber carbohydrate on microbial protein synthesis and dairy ration digestibility. Vet World.

[26] Yu S, Li L, Zhao H, Tu Y, Liu M, Jiang L (2023). Characterization of the dynamic changes of ruminal microbiota colonizing citrus pomace waste during rumen incubation for volatile fatty acid production. Microbiol Spect.

[27] Samadi, Feng X, Prates L, Wajizah S, Zulfahrizal, Munawar AA (2023). Effects of fermentation on protein profile of coffee by-products and its relationship with internal protein structure measured by vibrational spectroscopy. Anim Biosci.

[28] Putri EM, Zain M, Warly L, Hermon H (2021). Effects of rumen-degradable-to-undegradable protein ratio in ruminant diet on in vitro digestibility, rumen fermentation, and microbial protein synthesis. Vet World.

[29] Pu X, Guo X, Shahzad K, Wang M, Jiang C, Liu J (2020). Effects of dietary non-fibrous carbohydrate (NFC) to neutral detergent fiber (NDF) ratio change on rumen bacteria in sheep based on three generations of full-length amplifiers sequencing. Animals.

[30] Wang P, Tan Z (2013). Ammonia assimilation in rumen bacteria: a review. Anim Biotechnol.

[31] Mohammed AH, Shaikh TT (2023). Review on effect of the rumen protozoa on the productivity performance of some ruminant. Eur J Theor Appl Sci.

[32] He Y, Sun X, You P (2021). Animal, feed and rumen fermentation attributes associated with methane emissions from sheep fed brassica crops. J Anim Physiol Anim Nutr.

[33] Anas MA, Muhlisin, Bachruddin Z, Yusiati LM (2020). *In vitro* gas production kinetics as influenced by combination of *Acacia magium*
*Swietenia mahagoni*
*Artocarpus heterophyllus* as tannin source. IOP Conf Earth Environ Sci.

[34] Pu X, Zhang X, Yi S, Wang R, Li Q, Zhang W (2024). Mixed ensiling plus nitrate destroy fibre structure of rape straw, increase degradation, and reduce methanogenesis through in vitro ruminal fermentation. J Sci Food Agric.

[35] Morgavi DP, Forano E, Martin C, Newbold CJ (2010). Microbial ecosystem and methanogenesis in ruminants. Animal.

[36] Jayanegara A, Makkar HPS, Becker K (2015). Addition of purified tannin sources and polyethylene glycol treatment on methane emission and rumen fermentation *in vitro*. Media Peternakan.

[37] Elghandour MMY, Vázquez JC, Salem AZM, Kholif AE, Cipriano MM, Camacho LM (2017). *In vitro* gas and methane production of two mixed rations influenced by three different cultures of *Saccharomyces cerevisiae*. J Appl Anim Res.

[38] Sahroni WP, Permana IG, Despal (2021). Reformulation of dairy cow diets based on rumen degradable protein and total digestible nutrient with varying levels on *in vitro* fermentability and digestibility. IOP Conf Ser Earth Environ Sci.

[39] Hristov AN, Oh J, Firkins JL, Dijkstra J, Kebreab E, Waghorn G (2013). Special topics--mitigation of methane and nitrous oxide emissions from animal operations: I. A review of enteric methane mitigation options. J Anim Sci.

